# Clinical Insights into Physical Activity, Fitness, and Cardiovascular Health

**DOI:** 10.3390/jcm13195865

**Published:** 2024-10-01

**Authors:** Ina Volis, Barak Zafrir

**Affiliations:** 1 Department of Cardiology, Rambam Health Care Campus, Haifa 3109601, Israel; ina.volis@gmail.com; 2 Department of Cardiology, Lady Davis Carmel Medical Center, Haifa 3436212, Israel; 3 Faculty of Medicine, Technion, Israel Institute of Medicine, Haifa 3525433, Israel

A vast amount of data from numerous studies conducted in recent decades consistently show that increased physical activity has a favorable impact on cardiovascular health. The evidence for this beneficial effect arises from both subjective sources in the form of self-reported questionnaires and objective measures, including the assessment of cardiorespiratory fitness (CRF) using wearable devices and exercise cardiopulmonary tests [1,2]. Exercise testing provide valuable diagnostic and prognostic information regarding the risk of developing cardiovascular and pulmonary diseases [3]. The cardiopulmonary exercise test (CPET) offers insights into the patient’s physical capacity, fitness, and ability to perform daily activities, while allowing for the assessment of cardiorespiratory function and distinguishing between cardiac, ventilatory, and musculoskeletal limitations during exercise [4]. Moreover, the CPET helps to determine the appropriate exercise modes and intensity, as well as to monitor the effectiveness and safety of physical training interventions and therapeutic responses to cardiac device implantation [4].

Routine physical activity and exercise play a crucial role in both the primary and secondary prevention of cardiovascular diseases, showing an inverse, graded association with the degree of CRF. The cardioprotective effect of exercise, mediated by CRF, is believed to be affected by improvements in various modifiable cardiovascular risk factors, as well as improved psychosocial well-being [5]. Changes in CRF are associated with a proportional change in mortality risk for individuals both with and without cardiovascular disease, suggesting considerable clinical and public health implications [6]. The substantial body of evidence supporting the recommendations for an active lifestyle and regular physical activity is reflected in the guidelines for cardiovascular prevention, which provide unequivocal endorsement for routine physical activity for adults of all ages [5]. Nevertheless, with the rise of modernization, industrialization, and urbanization, a sedentary lifestyle and physical inactivity are increasingly common, affecting over a quarter of the adult population, and are associated with an increase in cardiometabolic comorbidities. A sedentary lifestyle leads to low CRF, which, in turn, is an independent risk factor for cardiovascular diseases, including coronary artery disease, heart failure, and mortality [2]. Despite the plethora of benefits of exercise on cardiovascular health and the significance of the CPET in the evaluation of various health and disease aspects, these tools are frequently underutilized in daily clinical practice. They are not commonly incorporated into the evaluation, treatment, and prevention of cardiovascular disorders, nor in risk stratification and prognosis prediction [4].

This Special Issue, titled “Clinical Insights into Physical Activity, Fitness, and Cardiovascular Health”, of the Journal of Clinical Medicine, comprises six papers addressing diverse topics in the field of CRF and sports cardiology, aiming to highlight clinical insights regarding exercise capacity, intensity, and various modalities in the context of cardiovascular health and disease. The degree of CRF can be objectively evaluated through treadmill exercise testing and serves as an independent prognostic marker, particularly for mortality [7]. Aker and colleagues (contribution 1) studied 6836 middle-aged individuals without pre-existing cardiovascular disease, categorizing their CRF levels based on their achieved treadmill exercise testing time and classifying them into age- and sex-specific quintiles. In their cohort, individuals with low fitness had a significantly higher risk of major adverse cardiovascular events (MACEs), including myocardial infarction, stroke, and all-cause mortality. Specifically, for every 1-MET (metabolic equivalent) decrease in peak exercise capacity, the risk of MACEs increased by 18%. Low fitness was associated with a high burden of cardiometabolic risk factors and independently predicted MACEs after multivariable adjustment. This emphasizes the critical role of lifestyle interventions in this population of middle-aged individuals without known cardiovascular disease. The simple and widely used treadmill exercise test is not typically employed for CRF evaluation but is mainly performed for the detection of cardiac ischemia. The study findings highlight the importance of CRF assessment using exercise testing as a prognostic marker, with a potential to improve predictive models of cardiovascular risk and assist in identifying high-risk populations for targeted primary prevention strategies.

Secondary prevention is another important aspect of physical activity. Exercise intolerance, with reduced capacity to perform physical activities accompanied by symptoms of dyspnea and fatigue [8], is a cardinal symptom of heart failure and is associated with a poor quality of life and increased mortality [9]. A study by Kisiel-Sekura and colleagues (contribution 2) explored the relationship between physical fitness, assessed through functional fitness tests (senior fitness test tasks), and exercise capacity, evaluated by the CPET, in men with heart failure with a reduced ejection fraction (HFrEF). The findings revealed that as the peak VO2 decreased, there was a corresponding decline in aerobic capacity, agility, muscle strength, and endurance, indicating that reduced exercise tolerance contributed to diminished physical function in this population. Limitations in physical fitness were particularly evident in patients exhibiting an excessive ventilatory responses to exercise (VE/VCO2 slope ≥ 35), even in those with preserved exercise tolerance (peak VO2 ≥ 18 mL/kg/min). This correlation could be useful for evaluating the overall functional and clinical status, as well as assessing risk in patients with HFrEF, especially those with low exercise capacity. The study underlines the essential role of the CPET in the clinical care of patients with heart failure.

The pathways and mediators through which physical activity and sports training benefit health are intriguing and have become a focus for both clinical and basic research. Matías Ruíz-Uribe and colleagues (contribution 3) investigated the metabolic effects of Moderate-Intensity Constant Training (MICT) and High-Intensity Interval Training (HIIT) in a small group of adults awaiting bariatric surgery. Their findings suggested that while both exercise regimens offered metabolic benefits, these were tissue-specific: MICT primarily improved skeletal muscle metabolism, whereas HIIT had a more pronounced impact on the liver and adipose tissue. The results of the study underscore the importance of selecting exercise regimens based on individual patient risk factors to achieve targeted metabolic outcomes.

Exercise training is a key component of cardiac rehabilitation, playing a crucial role in reducing the risk of recurrent events in patients who have experienced a cardiovascular event or undergone a cardiac intervention [10]. Traditionally, remote cardiac rehabilitation programs emphasize aerobic training. Unlike the conventional approach, a study conducted at Sheba Medical Center by Nabutovsky and colleagues (contribution 4) investigates the effects of a strength-training-focused regimen in a remote cardiac rehabilitation program. In this study, strength training significantly improved muscle endurance compared with the control group, who followed a standard aerobic-based program. Additionally, functional abilities and patient-reported physical health showed greater improvements in the strength training group. Importantly, the strength training group demonstrated higher adherence and engagement with the program. Both groups enhanced their CRF (measured by METs) in a repeat treadmill test, with no significant difference between them. These findings indicate that integrating strength training into remote cardiac rehabilitation can greatly enhance muscle endurance and patient engagement, providing a more comprehensive approach to rehabilitation. It points to a potential shift in remote cardiac rehabilitation practices, recommending the inclusion of strength training alongside aerobic exercises to improve patient outcomes.

The emphasis on physical activity and sports has resulted in the emergence of a new specialty—sports cardiology—which has evolved into a distinct clinical entity in recent years and is recognized worldwide as a subspecialty within cardiology [11]. Evaluation for exercise prescription, pre-participation screening of both competitive athletes and individuals engaged in recreational sports, as well as preventing sudden cardiac death and eligibility/disqualification for athletes with cardiac abnormalities, are among the practice areas of a sports cardiologist [12]. The European Society of Cardiology (ESC) guidelines classify sports according to their primary components—skill, power, mixed, and endurance—covering a diverse range of disciplines that include various isometric and isotonic exercises, each contributing uniquely to exercise-induced heart remodeling and adaptation. Both judo and weightlifting are categorized as power sports; however, a study by Di Gioia and colleagues (contribution 5) revealed notable differences in cardiac adaptations between Olympic judokas and weightlifters. Utilizing echocardiography and the CPET, they found that judokas had larger left and right ventricular dimensions and left atrial volumes, as well as increased left ventricular mass, compared with weightlifters. These findings suggest that judo, while classified as a power sport, incorporates more dynamic, aerobic elements, leading to distinct cardiac remodeling patterns compared with the purely anaerobic nature of weightlifting. Consequently, judo may be better categorized within a mixed classification rather than solely as a strength-based sport. Similarly to the cardiac rehabilitation programs focusing on aerobic training, the lifestyle recommendations for healthy non-athlete individuals are mainly aerobic in nature. A systematic review by Muñoz-Vásquez and colleagues (contribution 6) reviewed the impact of Olympic combat sports classified by the ESC guidelines as incorporating power components and inducing more anaerobic cardiac adaptation responses on CRF in non-athletes. The review analyzed randomized controlled trials involving participants of various ages, ranging from school children to older adults. It found that Olympic combat sports, such as taekwondo, karate, and boxing, significantly improved CRF via improvements in VO2 max, which is an important marker of cardiovascular health. Despite the benefits, the study emphasized that the quality of evidence was moderate to low due to study design limitations, inconsistent results, and high heterogeneity among the trials. The authors recommend further research with higher-quality methodologies to confirm these results.

In summary, regular physical activity, including structured exercise, is associated with reduced mortality and is an important component in the endeavors to prevent cardiovascular disease and its associated health consequences [5]. In the current sedentary era, in which leisure-time and occupation-related physical activity rates are consistently reduced, the promotion of regular physical activity and exercise is of vital importance and should be emphasized in any encounter in clinical practice between patients and healthcare personnel. The growing field of sports cardiology, as well as tele-monitoring and tele-rehabilitation, will advance the vision of “Exercise Is Medicine” to make physical activity assessments and exercise prescription a standard part of disease prevention and treatment plans for all patients [13]. A solid scientific background is needed to overcome knowledge gaps and provide evidence-based recommendations, aiming to integrate fitness into preventive healthcare. Just as we prescribe medications based on our patients’ conditions or diagnoses, we should also aim to prescribe physical activity for them, whether for primary or secondary prevention. Exercise should be customized to each individual, considering their existing health issues, risk factors, and capabilities. We must work toward positive interventions across all areas of prevention for our patients, not solely through medications. In order to effectively tailor different types of physical activity and create precise exercise plans, additional research is needed regarding the mechanistic and physiological effects of various sports on cardiovascular health.
